# Budding and Division of Giant Vesicles Linked to Phospholipid Production

**DOI:** 10.1038/s41598-018-36183-9

**Published:** 2019-01-17

**Authors:** Juan M. Castro, Hironori Sugiyama, Taro Toyota

**Affiliations:** 1 Department of Basic Science, Graduate School of Arts and Sciences, The University of Tokyo, 3-8-1 Komaba, Meguro-ku, Tokyo, 153-8902 Japan; 2 Universal Biology Institute, The University of Tokyo, 3-8-1 Komaba, Meguro-ku, Tokyo, 153-8902 Japan

## Abstract

The self-reproduction of supramolecular assemblies based on the synthesis and self-assembly of building blocks is a critical step towards the construction of chemical systems with autonomous, adaptive, and propagation properties. In this report, we demonstrate that giant vesicles can grow and produce daughter vesicles by synthesizing and incorporating phospholipids *in situ* from *ad-hoc* precursors. Our model involves acyl chain elongation via copper(I)-catalyzed azide-alkyne [3 + 2] cycloaddition reaction and the ensuing production of synthetic phospholipids to induce budding and division. In addition, the growth and budding of giant vesicles were compatible with the encapsulation and transfer of macromolecules as large as lambda phage DNA to the buds. This chemical system provides a useful model towards the implementation of cell-like compartments capable of propagation and transport of biological materials.

## Introduction

Self-reproduction is central to the propagation and evolutionary adaptation of life. New cells are created only when preexisting cells grow and divide. The generation of simple supramolecular structures capable of growth and division can, thus, contribute to our understanding of primitive self-reproduction and to the construction of systems with autonomous, adaptive, and propagation properties^1–6^. During the past 50 years, giant phospholipid vesicles have been used as models for the membranes of biological cells. Essential cellular behaviors, such as growth and division, have been studied and developed under relatively simple conditions. For instance, fission of giant vesicles (GVs) was demonstrated using external stimuli that include temperature variations^7,8^ mechanical stress^9^, and changes in phase separation^10^. It has also been proved that shape transformations and division of GVs can be induced by vesicle fusion or external addition of membrane additives^11–14^. In parallel, various studies have explored the use of chemical induction to generate membrane growth and division in the presence of reactive membrane precursors. Hydrolysis of carboxylic acid anhydrate^15^, imine bond formation and decomposition^16–18^, triazole formation by copper(I)-catalyzed azide-alkyne [3 + 2] cycloaddition^19^, and thioester exchange reaction^20^ have been adopted for the construction of synthetic amphiphiles in order to facilitate these processes.

Although shape transformations and fission in GVs have been demonstrated using various methods, the capability to control these processes to couple budding and growth and afford spherical, single-walled daughter vesicles with diameters of approximately 5 μm through division remains limited. On the other hand, compartmentalization and transport of macromolecules and reagents within self-reproducing systems are critical requirements for the synthesis of dynamic microreactors and model protocells. In vesicular systems, this step has been limited in part by the size and inner structure of the mother and daughter vesicles. Self-reproducing GVs with large diameters, spherical shape, and simple membrane structure are desirable not only to facilitate the encapsulation and transport of different materials; but also to enable direct visualization of the encapsulated molecules by fluorescence microscopy and fluorescent probes.

**Figure 1:**
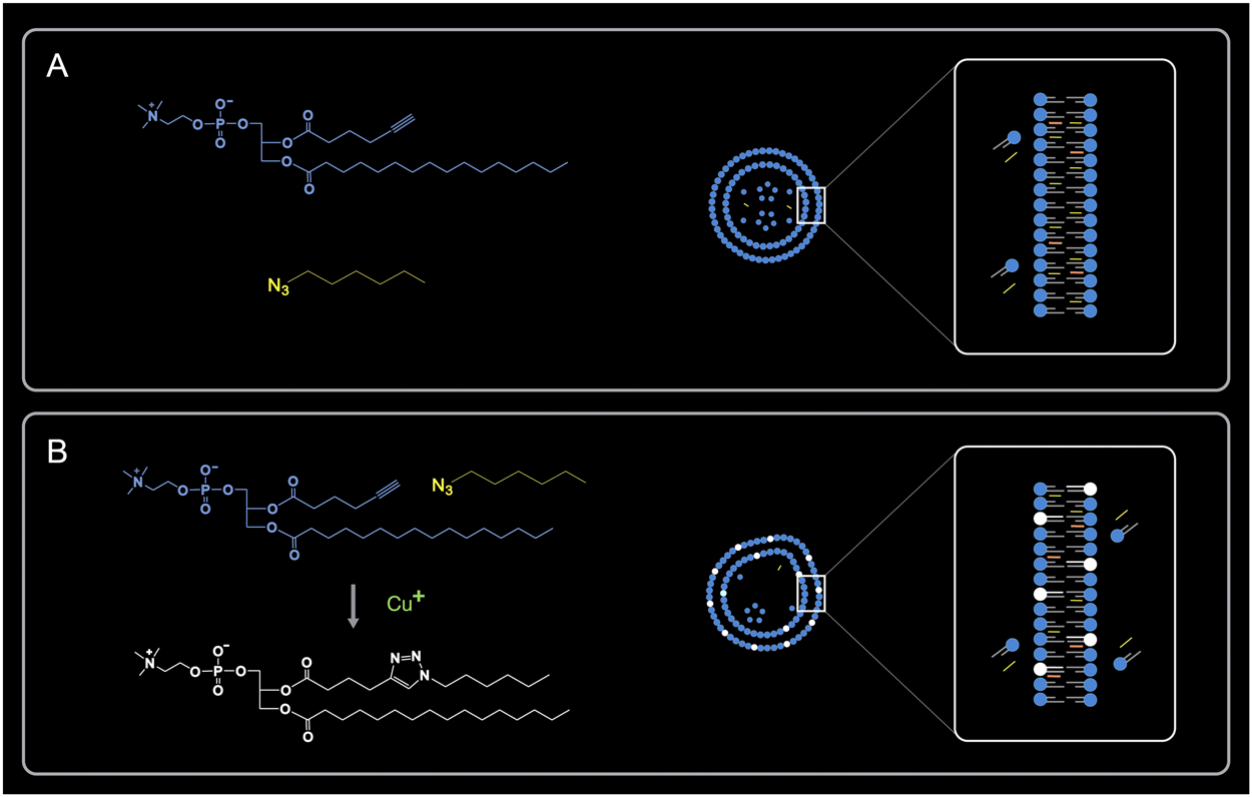
Schematic illustration showing the transformation of an immature mother into a mature mother. (**A**) Left: Chemical structures of *ad-hoc* precursors LH (blue) and AH (yellow). Right: representation of an immature mother composed of LH (blue), AH (yellow), and 5 mol% of cholesterol (orange). (**B**) Left: chemical structure of TPC (white) formed by LH and AH in the presence of copper(I) ion via the CuAAC reaction. Right: representation of a mature mother upon chemical conversion and the formation of TPC. TPC becomes integrated into the GV membrane determining its own growth, budding, and division.

In this report, we demonstrate that GVs can grow and produce daughter vesicles by synthesizing and incorporating phospholipids *in situ* from *ad-hoc* precursors. We approached the problem of self-reproduction by constructing a minimal vesicular system whose membrane acts both as a stable compartment and as a responsive phospholipid-forming interface. Specifically, we designed and synthesized a short-chain alkyl azide (AH) and an alkyne amphiphile (LH) to induce acyl chain elongation of the latter using the copper(I)-catalyzed azide-alkyne [3 + 2] cycloaddition (CuAAC) reaction^19,21–23^. We chose a short-chain alkyl azide to allow greater dispersion of alkyl azides in water compared to previously reported techniques^19,23^. Upon addition of a catalytic solution and *ad-hoc* precursors, this vesicular system readily forms a synthetic phosphatidylcholine containing a triazole ring at its hydrophobic chain (TPC). This additional membrane component becomes integrated into the GV membrane, determining its own growth, budding, and division. Herein, we refer to the GVs composed of LH and AH before chemical induction as “immature mothers” and the chemically active, phospholipid-forming GVs in the presence of a CuAAC catalyst as “mature mothers” (Fig. 1). In addition, we found that growth and budding are compatible with the transfer of biological materials, such as DNA, to the buds. Owing to their size (approximately 5 μm in diameter) and nominal single-walled structure, macromolecules as large as lambda phage DNA (48 kb) were encapsulated within the immature mother GVs and later observed in the buds.

## Results

### Immature mother GVs composed of *ad-hoc* precursors

The addition of alkyne- or azide-functional moieties in the molecular structure of amphiphiles coupled with the formation of triazoles provide various opportunities for functionalization and modification of vesicular surfaces^24–26^. These synthetic amphiphiles can be incorporated in lipid bilayers and further reacted with complementary molecules without damaging the vesicles^27,28^. Moreover, in the synthesis of 1,2,3-triazoles, the CuAAC reaction is an effective chemoselective and water-tolerant ligation tool that can be used for synthesizing stable cycloaddition products, such as novel phospholipids, to induce growth and splitting of vesicles^19,23^. In living cells excess membrane synthesis is sufficient to produce shape transformations and proliferation. Endogenous phospholipid production has been suggested as the key factor in L-form cell shape changes and division^29–31^. Accordingly, we reasoned that a synthetic GV composed primarily of *ad-hoc* precursors drives bilayer synthesis via a simple catalytic reaction and the presence of complementary reactive molecules by a continuous variation between phospholipid production and available surface area, leading to growth and daughter formation. We therefore aimed to develop a minimal vesicular system to investigate *in situ* membrane molecule synthesis^32^.

The GVs that we developed consist of two types of synthetic molecules. The first, lysolecithin esterified with hexynoic acid (LH), is an alkyne-functional amphiphile. The second, 1-azidohexane (AH), is a short-chain alkyl azide. LH and AH were used in the presence of a small amount of cholesterol (Chol) as a membrane reinforcing factor (Fig. 1A). After the addition of a copper catalyst, the cycloaddition induces the acyl chain elongation of LH and the resulting production of a synthetic phospholipid with a triazole ring, TPC (Fig. 1B).

We prepared GVs consisting of LH, AH, and Chol (4.5/4.5/0.5 mM) by lipid dispersion mixture. Briefly, an aqueous dispersion of LH (9 mM) and a mixture of AH (9 mM) and Chol (1 mM) were prepared separately and then equal volumes were mixed for 10 h at room temperature. We found that without cholesterol in the lipid dispersion, neither LH or AH produce GVs. Stained GVs were prepared by mixing stock solutions of AH/Chol and sulforhodamine 101-1,2-dihexadecanoyl-*sn*-glycero-3-phosphoethanolamine, triethylammonium salt (TexasRed-DHPE, 0.02 mol%). Microscopy examination of samples revealed the presence of numerous GVs with a diameter >10 μm (Supplementary Figs S1, S2). Transmission electron microscopy shown that the vesicular membranes have a thickness of 2–3 nm and are stacked or partly fused (Supplementary Fig. S3).

In order to confirm the lipid composition of the GVs before chemical induction, we analyzed the dispersions of GVs using ^1^H nuclear magnetic resonance (NMR) spectroscopy. We examined the dispersions of GVs 20 min and 10 days after their preparation. The chemical analysis of these dispersions revealed small amounts of TPC (Supplementary Table S1). The sample analyzed 20 min after its preparation revealed an amount of TPC of approximately 8 mol% of total lipids. Accordingly, these GVs were termed “immature mothers” because without a catalyst and chemical feeding they were not capable of growth, budding, or division processes (described in more detail later).

### Growth, budding, and division of mature mothers

**Figure 2:**
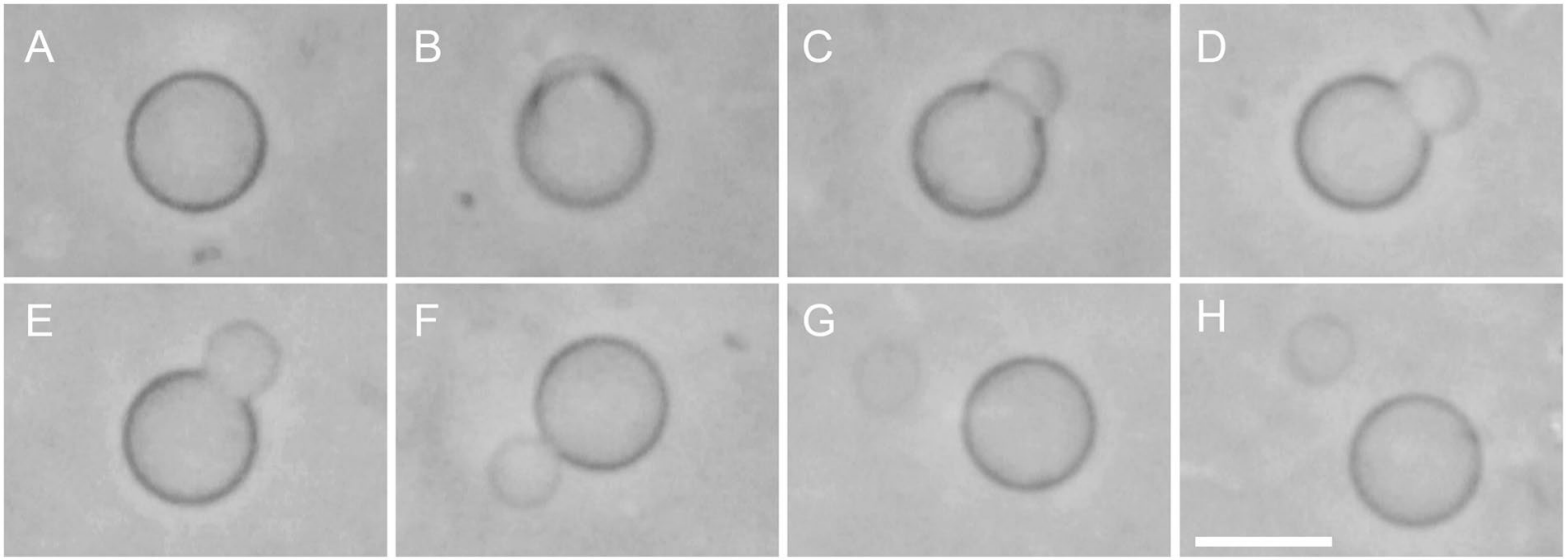
Budding and division of a mature mother GV driven by the addition of a catalytic solution and reactive precursors. Selected frames from a phase contrast microscopy video (Supplementary Movie S1). The first image (**A**) was taken 15 min after the mixing of equal volumes of a dispersion of immature mothers, a catalytic solution (copper (I) chloride (10 mM), ascorbic acid (20 mM), and deionized water) and lipid dispersions of precursors LH (9 mM) and AH/Chol (9/1 mM). Images were acquired at: (**A**) 15:08 sec, (**B**) 16:52 sec, (**C**) 22:09 sec (**D**) 24:03 sec, (**E**) 26:04 sec, (**F**) 30:09 sec, (**G**) 34:28 sec, (**H**) 45:07 sec. Scale bar, 10 μm.

Copper (I) chloride was used as the catalyst of the CuAAC reaction in water^19,23^. Ascorbic acid is an established antioxidant, which reduces the Cu(II) ions produced by the autoxidation of Cu(I) in aqueous solution^33,34^. Consequently, we added ascorbic acid to the copper (I) chloride aqueous solution to maintain the catalytic effect of Cu(I). Following the addition of a catalytic solution (copper (I) chloride (10 mM), ascorbic acid (20 mM), and deionized water) and reactive precursors (LH, 9 mM; AH/Chol, 9/1 mM), we observed the membrane transformation of the GVs. Phase contrast and fluorescence microscopy of the GVs on glass slides revealed budding and vesicle formation followed by division (Fig. 2 and Supplementary Movie S1). Initially, the spherical immature mothers began to form buds and deformed into pear-like structures with a wide neck (Supplementary Fig. S4). This budding formation typically occurred within 10–25 min. Subsequently, the buds became spherical vesicles. For a short period of time, the spherical buds remained connected to the mature mothers by a thin neck. Eventually, the neck disappeared and the newborn “daughter” GVs separated from the mothers. Fluorescence microscopic images of the GVs during budding revealed an asymmetrical distribution of the TexasRed-DHPE fluorescent dye between the mother vesicles and the spherical buds (Supplementary Fig. S5). In the same way, phase contrast microscopy and video sequences shown that the contrast of the pear-like buds was weaker than that of the mothers (Fig. 2 and Supplementary Movie S1). In addition to the spherical mature mothers, we occasionally observed the budding and division of non-spherical mature mothers (Supplementary Fig. S6). In total, we conducted the observation of mature mothers in presence of both the catalytic solution and precursors 26 times. Each of these experiments was performed on a different day. Using the same experimental conditions, we observed the GVs showing budding and following division 17 times.

As a reference experiment, we examined whether these processes could still occur without the addition of copper (I) chloride or reactive precursors. Firstly, we studied the transformation of the immature mothers using lipid dispersions of LH (9 mM) and AH/Chol (9/1 mM) without the catalytic solution. For this experiment, we replaced the catalytic solution with deionized water. Immature mothers on a glass slide did not display appreciable change in size or morphology during 2 h following the addition of deionized water and reactive precursors. In the presence of the catalytic solution without the reactive precursors, the GVs displayed deformations of the outer layer during the first 5–10 min. However, after 15 min, they resumed their spherical shape.

Secondly, to elucidate the role of the *ad-hoc* precursors, we performed several reference experiments by replacing the catalytic solution to examine whether the budding and division processes occurred or changed. Initially, we studied the transformation of the immature mothers with an electrolyte solution of another cation with a chloride anion in the absence of precursors. In this case, the GVs were subjected to sodium chloride (10 mM), ascorbic acid (20 mM), and deionized water (non-catalytic solution) instead of the catalytic solution with copper (I) ions. In one specimen observed 10 min later the GVs were often bordered by various small buds. Ultimately, this activity was accompanied by multiple membrane ruptures and the collapse of the vesicle compartments after 15–30 min (Supplementary Fig. S7). Finally, we analyzed the transformation of the immature mothers using lipid dispersions of LH (9 mM) and AH/Chol (9/1 mM) with the non-catalytic solution (sodium chloride, 10 mM; ascorbic acid, 20 mM; and deionized water). After the addition of the lipid dispersions and the non-catalytic solution, the GVs gradually shrunk and formed thick lipid aggregations within them. After 15–30 min, the GVs collapsed at the surface of the glass slide (Supplementary Fig. S8). These reference data indicated that the addition of copper (I) chloride (with ascorbic acid) and membrane precursors facilitated the budding and division of the mature mother GVs.

To determine the lipid composition of the mother GVs and the extent of the CuAAC reaction, we examined the dispersion of GVs in the presence of the catalytic solution and membrane precursors by ^1^H NMR spectroscopy. The CuAAC reaction was allowed to proceed for 1 h at room temperature (23–25 °C). When we added the catalytic solution to the dispersion of immature mothers, the amount of TPC increased to approximately 13 mol% of the total lipids (Supplementary Table S1). Moreover, the production of TPC reached approximately 30 mol% when the GVs were fed with precursors (LH and AH/Chol) in presence of copper (I) chloride and ascorbic acid. These data indicated that the dispersion of GVs in the presence of the catalytic solution and precursors produced TPC during the budding and following division of the GVs via the CuAAC reaction.

**Figure 3:**
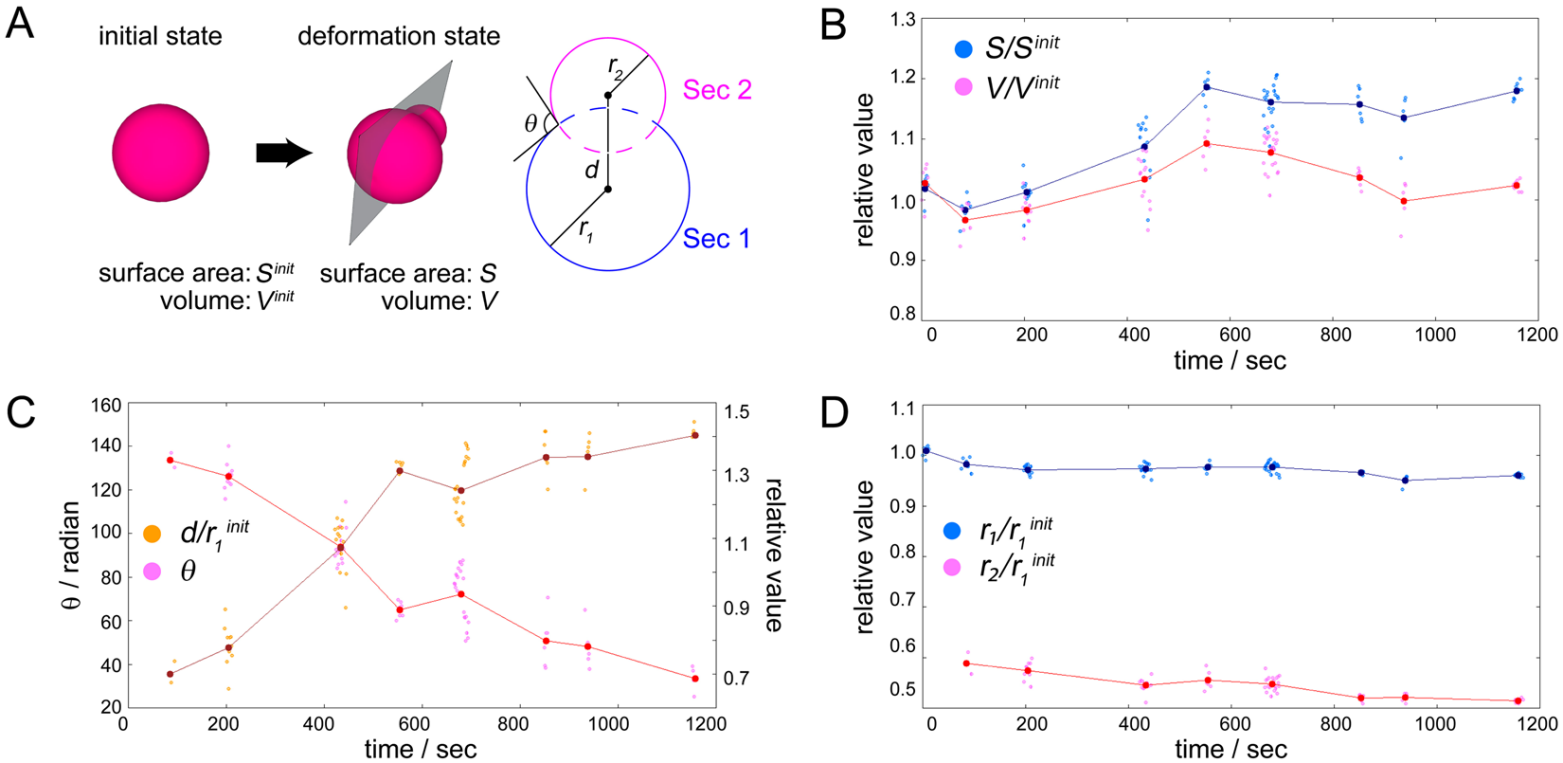
Time course of relative volume and surface area of a mature mother GV during budding (Fig. 2 and Supplementary Movie S1) and cross-section geometrical parameters. (**A**) Illustration of the cross-section and analyzed parameters. (**B**) Plot of the average values of relative surface area (blue) and volume (magenta) of an individual mother GV during budding. (**C**) Plot of the angle (red dots) and the distance of the centers of Sec. 1 and Sec. 2 (brown dots). (**D**) Plot of the relative radii of Sec. 1 (blue) and Sec. 2 (magenta). The initial radius of Sec. 1 was assigned as 1 for these relative radii. The pale-colored dots represent all the data obtained by the analysis and the intensely colored dots represent the average of the data set form snapshots taken from one movie.

We next sought to verify by image analysis if the budding and division processes were induced by membrane growth. Accordingly, we analyzed several video sequences of mother GVs recorded during microscopy using a custom-made software described in the Methods section. Typical spherical mothers of 7 to 10 μm in diameter were used for the analysis. Non-spherical mothers were omitted because it was difficult to make precise image approximations of these three-dimensional objects and their membrane fluctuations. The normalized surface area and volume of the mothers that displayed budding were calculated. For the analysis, the mother GVs were assumed to be rotationally symmetric and it was also assumed that the two-dimensional images (cross-section) of the budding GVs with a pear-like structure and wide neck could be fitted as two superimposed circles (Fig. 3A). The analytical results of the shape transformation of a mother GV (Supplementary Movie S1) are shown in Fig. 3. The relative surface area of this mature mother increased from 1 to approximately 1.2 within the first 600 secs (Fig. 3B). The time required for complete division after budding was 1200–1300 sec. Next, we used the Mann-Whitney’s U test to statistically verify the difference between these two tendencies. This nonparametric test was chosen owing to the uncertainty of the noise distribution. The first and final data sets of the relative surface area of the mother GV during budding were statistically distinguishable, while, in the case of relative volume, the first and final data sets were statistically indistinguishable. In a different video sequence (Supplementary Movie S2, Fig. S9), the same tendency was obtained. Hence, in terms of growth rate, the results suggested that in these examples there is a different tendency among relative surface area and relative volume during the budding process.

To further characterize the budding process of the mature mothers, we utilized image analysis of cross sections of the GVs focusing on their pear-like structures and wide necks. Phase contrast microscopy images of the GVs were divided into two fan-shaped sections (Sec. 1 and Sec. 2 in Fig. 3A). We measured the angle *θ* between two tangents at the neck, the distance *d* between the centers of Sec. 1 and Sec. 2 (Fig. 3C), and their radii (Fig. 3D). Throughout the budding process, continuous change of the curvature at the neck was maintained. Moreover, the time courses of angle *θ* and distance *d* showed a clear negative correlation. In addition, the radius of Sec. 1 displayed a shrinking tendency during the budding process. These analytical results indicated that the buds do not form in the vicinity of the mother GVs surface, but rather are directly linked to the mother GVs membrane and the transfer of lipid molecules from the mother to their buds.

### Encapsulation and transport of lambda phage DNA

To test the encapsulation and transport properties of our vesicular system, we prepared immature mother GVs and encapsulated DNA molecules. For this procedure, we chose commercially available lambda phage DNA because of its large genome size (48 kb) and supercoiled structure. Before encapsulation, these molecules were labeled with SYBR Green I fluorescent dye for microscopic visualization. Fluorescence microscopy confirmed that lambda phage DNA was encapsulated within the immature mother GVs during *in situ* self-assembly (Supplementary Fig. S10).

**Figure 4:**
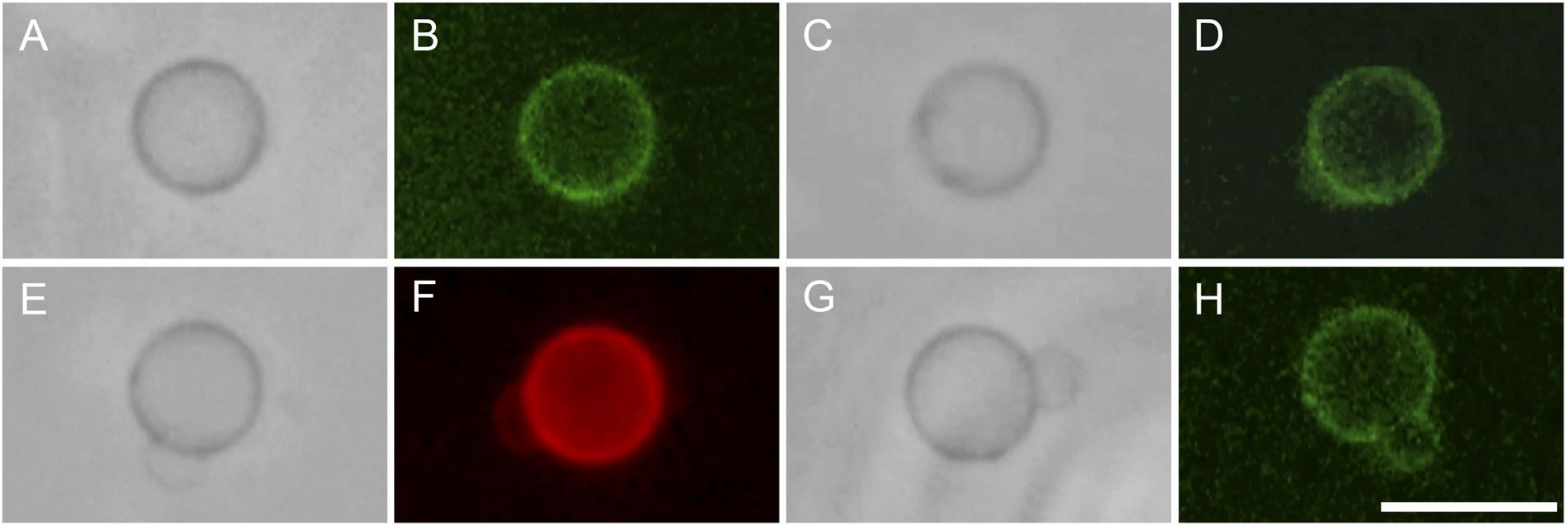
Localization and transport of lambda phage DNA molecules from a mature mother to its bud. Phase contrast and fluorescence micrographs of a GV labeled with 0.02 mol% TexasRed-DHPE and encapsulated DNA stained by SYBR Green I. The first image was taken 13 min after the mixing of a dispersion of DNA-containing immature mothers, a catalytic solution (copper (I) chloride, 10 mM; ascorbic acid, 20 mM; and deionized water) and reactive precursors LH (9 mM) and AH/Chol (9/1 mM) (*t* = 0 sec). Images were acquired at: (**A**) 13:03 sec, (**B**) 18:23 sec, (**C**) 20:01 sec, (**D**) 20:29 sec, (**E**) 22:06 sec, (**F**) 23:13 sec, (**G**) 35:14 sec, (**H**) 38:18 sec. Scale bar, 10 μm.

Next, we mixed a dispersion of DNA-containing immature mothers with the catalytic solution and dispersions of membrane precursors. Interestingly, fluorescence microscopy revealed that immediately after mixing, the encapsulated DNA molecules rapidly accumulated in the vicinity of the GVs membrane (Fig. 4 and Supplementary Movie S3). Concerning the morphological transformations, growth and budding consistently occurred within 10–30 min. We observed gradual growth and the deformation of the GVs into pear-like structures. Finally, we observed partial division with the buds remaining connected to the mothers and the transfer of the DNA molecules to the bilayers of the buds. The fluorescence intensity profiles of the GVs demonstrated that DNA was localized in the membrane of both the mother and buds (Supplementary Fig. S11).

To clarify the accumulation of DNA in the membrane of the GVs, three reference experiments were performed. In the first, we exposed the DNA-containing GVs to membrane precursors without the catalytic solution for 1 h. DNA remained visible in the entire inner space of the vesicle without variation, no relocation of lambda phage DNA was evident (Supplementary Fig. S12). In the second experiment, we omitted DNA during encapsulation. In this case, the immature mother GVs were exposed to the SYBR Green I solution and the Tris-EDTA buffer instead of lambda phage DNA. As expected, green fluorescence was not detected under the same observation conditions including magnification and numerical aperture of the objective lens, irradiation intensity, exposure time of the mercury lamp, and temperature. This indicated that SYBR Green I alone did not fluoresce in the lipid membrane of the GVs (Supplementary Fig. S13). In the third experiment, we encapsulated water-soluble Rhodamine B-tagged dextran instead of DNA. In the presence of the catalytic solution and the precursors, we observed budding, but there was no accumulation of the fluorescent molecule in the membrane of the mother or its bud (Supplementary Figs S14 and S15). The results of the experiments demonstrated that in the presence of the catalytic solution SYBR green I labeled-DNA was first relocated at the vicinity of the mother membrane and later, during growth and budding, it was transferred to the membrane of the bud.

## Discussion

The collective results strongly suggest that the growth, budding, and division of the mature mothers GVs can be directly attributed to the addition of the catalytic solution and membrane precursors. ^1^H NMR and image analyses demonstrated that these dynamics are linked to phospholipid synthesis and the production of additional membrane surface. It should be noted that the buds of the mature mothers mostly had a wide neck structure, as well as a pale appearance in both phase contrast and fluorescence micrographs. We consider the reason of these features as follow. The immature mothers have nominally a single outer membrane. However, this membrane comprises several sets of bilayers which can be assigned to an interdigitated state because of their thickness^35^. Upon addition of the reactive membrane precursors (LH and AH/Chol) and the catalytic solution to the immature mothers, external precursors and copper (I) ions incorporate into the GVs and interact with the membrane, i.e. LH and AH react with each other to produce TPC via the CuAAC reaction (Fig. 1). This brings about an increase in the number of synthetic phospholipids in the outer membrane. Since the interdigitated bilayers likely interconnect to each other through transient fusion^36^, the produced TPC diffuse into the bilayers comprising the outer membrane of the mothers. Note that TPC is a synthetic phospholipid with two acyl chains and a triazol moiety. Thus, the bilayers containing TPC gradually lose their dynamic interconnection and a biphasic membrane with a TPC-rich phase and an LH/AH-Chol phase is formed. The image analysis revealed that approximately 10 min after the addition of the catalytic solution and the precursors, the radius of Sec. 2 was almost constant or increased during budding (Fig. 3 and Supplementary Fig. S9). This was likely caused by the different spontaneous curvatures of LH (with AH and Chol) and TPC. Their distribution is also likely to form asymmetric GVs affecting the cholesterol-dependent fluidity and/or membrane tension^37–39^. We suppose that the lipid composition of the buds was comprised mainly of TPC with low concentrations of AH/Chol. Concerning the pale appearance of the buds, in the bilayers comprising the outer membrane of the buds TPC plausibly suppress the interconnection of the bilayers, resulting in the budding process and an outer membrane composed of less bilayers. Likewise, the TexasRed-DHPE fluorescent moiety was probably quenched or optically changed in the vicinity of the membrane because it contains condensed triazole rings^40,41^. The amount of TexasRed-DHPE in the buds was regulated from the mother GVs. Thus, the buds may not contain sufficient amounts of the fluorescent probe because of differing membrane fluidity. On the other hand, while the relative surface area of the budding mother GVs increased, their relative volume did not. Note that the aqueous environment outside the budding mother GVs was hypertonic due to the catalytic solution (the concentration of precursors was the same as that of the initial dispersion of the GVs). Thus, the initial osmolarity owing to pre-encapsulated LH and AH/Chol of the immature mothers may be another driving force for volume retention during budding. In a few cases, the budding GVs increase their relative surface area and displayed a decreased relative volume, implying that the osmolarity of the GVs may have been insufficient at the initial state (Supplementary Movie S4, Figs S16 and S17).

Although we observed this asymmetrical distribution of lipid components between the buds and the mother GVs, in the case of the mothers with encapsulated lambda phage DNA, there was a symmetric distribution of the DNA molecules in both vesicles. The buds and mother GVs exhibited a highly localized fluorescence in their membranes (Supplementary Fig. S11). This relocation of DNA after the addition of the catalytic solution and *ad-hoc* precursors suggests that there is an electrostatic interaction between the membrane and DNA through copper (I) ions. These observations are consistent with the condensation of DNA molecules in lipid vesicles composed of cationic lipids due to electrostatic attraction^42,43^. The current chemical system enables us to suggest that, in association with lipid production, copper (I) ions can penetrate into the GVs membrane (probably because of complexation with phosphate and/or triazole moieties of LH and TPC) and also deliver large DNA molecules to the buds.

In summary, we have shown that giant phospholipid vesicles composed of synthetic molecules can undergo growth and budding followed by daughter vesicle formation, which mimics cell growth and division. This was possible by exploiting phospholipid synthesis and the production of additional membrane surface via the CuAAC reaction. The model GVs are not only capable of growth, budding and division, but also of compartmentalization and transport of macromolecules, as demonstrated by the transfer of lambda phage DNA from a mature mother to its bud.

Growth of living cells crucially involves the synthesis of cell membrane lipids. The continuous production of lipid molecules is a key activity for self-maintenance, growth, and reproduction. Knowledge of the process of GV division in connection to phospholipid synthesis and the production of additional membrane surface represent a promising basis for the implementation of self-sustained reproduction. Although this study focused on a rather simple chemical system, it serves as a minimal and useful model towards the implementation of cell-like compartments capable of propagation and delivery of reagents.

## Methods

### Materials

L-α-Lysophosphatidylcholine,*N*,*N*-dicyclohexylcarbodiimide (DCCI), 4,4′-dimethylamin-pyridine (DMAP), cholesterol, and dichloromethane (CH_2_Cl_2_) were purchased from Wako Pure Chemical Industries, Ltd. (Tokyo, Japan). 5-Hexynoic acid was purchased from Tokyo Chemical Industry Co. (Tokyo, Japan). Silica gel (60/200) was purchased from Kanto Chemical Co. (Tokyo, Japan). Sulforhodamine101-1,2-dihexadecanoyl-*sn*-glycero-3-phosphoethanolamine, triethylammonium salt (Texas Red-DHPE) and RhodamineB-tagged dextran (zwitterionic, molecular weight = 10000) were purchased from ThermoFisher Scientific, Inc. (Waltham, MA, USA). Tris-EDTA Buffer 10x powder (pH 7.4) was purchased from Takara Bio Inc. (Shiga, Japan). Lambda phage DNA (48502-base pair) methylated from *Escherichia coli* host strain W3110 was purchased from Sigma-Aldrich (St. Louis, MO, USA). SYBR® Green I was purchased from LONZA (Basel, Switzerland). Water was distilled and deionized before use with a MilliQ system from Millipore (Billerica, MA, USA). All reagents were used as received. L-α-Lysophosphatidylcholine was an almost equal amount mixture of 3-(palmitoyloxy)-2-hydroxypropyl (2-(trimethylammonio)ethyl) phosphate, and 3-(stearoyloxy)-2-hydroxypropyl (2-(trimethylammonio)ethyl) phosphate, which was determined by electrospray ionization mass spectrometry using a model LCMS-2020 device (Shimadzu, Kyoto, Japan).

### Synthesis of lysolecithin esterified with hexynoic acid (LH) (mixture of 3-(palmitoyloxy)-2-(hexy-5-ynoyloxy)propyl (2-(trimethylammonio)ethyl) phosphate and 3-(stearoyloxy)-2-(hexy-5-ynoyloxy)propyl (2-(trimethylammonio)ethyl) phosphate

LH was synthesized as previously described^19,44,45^. CH_2_Cl_2_ (5 mL) was used as the reaction solvent. It was dehydrated using molecular sieves and transferred to a 100 mL round-bottom flask equipped with a mechanical stirrer. While the solvent was maintained at room temperature (23–25 °C) under an argon gas atmosphere, 5-hexynoic acid (88 μL, 0.8 mmol) was added using a micropipette and the reaction mixture was stirred for 5 min. DCCI (256 mg, 1.2 mmol) was then added. This resulted in a turbid reaction mixture. DMAP (49 mg, 0.4 mmol) was added and the reaction mixture was stirred for 10 min. After this step, L-α-lysophosphatidylcholine (100 mg, approximately 0.2 mmol) was added. Finally, the reaction mixture was stirred at room temperature overnight (20 h). After vacuum filtration of the reaction mixture to eliminate the precipitate, the solvent was removed under reduced pressure. The crude product was transferred with 5 mL of CH_2_Cl_2_ to a glass column (3 cm in diameter and 8 cm in length) packed with 13 g silica gel (equilibrated by chloroform). The product was purified by the sequential addition of 120 mL chloroform, 500 mL of 95:5 chloroform: methanol (v/v), 500 mL of 92:8 chloroform:methanol, 200 mL of 90:10 chloroform: methanol, 300 mL of 80:20 chloroform: methanol, and then collected by the addition of 300 mL of methanol. After the solvent was evaporated, this procedure yielded a pale brown paste (76 mg) of LH.

^1^H NMR (500 MHz, dimethylsulfoxide [DMSO]-*d*_6_): δ 5.09-5.05 (m, 1 H), 4.29–4.27 (dd, *J* = 11.9 Hz, *J* = 3.2 Hz, 1 H), 4.11–4.08 (dd, *J* = 11.9 Hz, *J* = 7.2 Hz, 1 H), 4.04–3.98 (m, 2 H), 3.73 (t, *J* = 5.8 Hz, 2 H), 3.51–3.49 (m, 2 H), 3.13 (s, 9 H), 2.80 (t, *J* = 2.7 Hz, 1 H), 2.39 (t, *J* = 7.3 Hz, 2 H), 2.27 (t, *J* = 7.4 Hz, 2 H), 2.20 (dt, *J* = 7.2 Hz, *J* = 2.7 Hz, 2 H), 1.70 (quin, *J* = 7.2 Hz, 2 H), 1.53–1.47 (t, *J* = 7.5 Hz, 2 H), 1.29–1.16 (br, 24–26 H), 0.85 (t, *J* = 6.9 Hz, 3 H).

### Synthesis of 1-azohexane (AH)

The synthesis protocol has been previously described^46^. Briefly, a DMSO solution (50 mL) of sodium azide (1.7 g, 27 mmol) was prepared at 40 °C. 1-Bromohexane (3.5 mL, 25 mmol) was added to the solution and the reaction mixture was stirred at 25 °C for 1 day. Distilled water (100 mL) was added to the reaction mixture and the mixture was cooled to room temperature. The crude product was extracted using 100 mL of diethylether three times. Then it was rinsed with distilled water (100 mL) followed by saturated NaCl aq. (100 mL). The product was dried by magnesium sulfate and filtered for purification. Finally, the solvent was removed under reduced pressure to afford a transparent liquid (yield 76%, 2.5 g).

^1^H NMR (270 MHz, chloroform-*d*_1_): δ 3.42 (*t*, *J* = 7.7 Hz, 2 H), 1.86 (t, *J* = 8.0 Hz, 2 H), 1.46–1.30 (m, 6 H), 0.89 (*t*, *J* = 6.2 Hz, 3 H).

### Synthesis of triazole-ring containing phosphatidylcholine (TPC): mixture of 3-(heptadecanoyloxy)-2-((3-(1-hexyl-1H-1,2,3-triazol-4-yl)hexaoyl)oxy)propyl (2-(trimethylammonio)ethyl) phosphate and 3-(stearoyloxy)-2-((3-(1-hexyl-1H-1,2,3-triazol-4-yl)hexaoyl)oxy)propyl (2-(trimethylammonio)ethyl) phosphate with a trace amount of copper ions

AH (8.0 mg, 0.06 mmol), LH (27 mg), and copper (I) chloride (3.0 mg, 0.03 mmol) were dissolved in methanol (10 mL) and reacted for 15 h at room temperature (23–25 °C) under an argon gas atmosphere. The reaction mixture was filtered to remove the precipitate. Finally, the solvent and remaining AH were removed under reduced pressure with an oil rotary pump. The residue was a pale green paste (33 mg).

^1^H NMR (500 MHz, methanol-*d*_4_): δ 7.93–7.85 (m, 1 H), 5.30–5.25 (m, 1 H), 4.45 (dd, *J* = 12.2 Hz, *J* = 3.4 Hz, 1 H), 4.38 (t, *J* = 7.0 Hz, 2 H), 4.33–4.27 (m, 2 H), 4.22 (dd, *J* = 12.2 Hz, *J* = 6.7 Hz, 1 H), 4.07–4.01 (m, 2 H), 3.70–3.64 (m, 2 H), 3.25 (s, 9 H), 2.81–2.74 (m, 2 H), 2.47–2.41 (m, 2 H), 2.33 (t, *J* = 7.4 Hz, 2 H), 2.06–1.98 (m, 2 H), 1.95–1.88 (m, 2 H), 1.64–1.57 (m, 2 H), 1.38–1.26 (m, 24–26 H), 0.91 (t, *J* = 7.0 Hz, 6 H).

### Preparation of giant vesicles

L-α-Lysophosphatidylcholine esterified with hexynoic acid (9 mM) was dissolved in chloroform using an amber glass vial. A thin film was formed by the evaporation of the solvent. The residual chloroform was removed by placing the glass vial under reduced pressure for approximately 12 h. After the addition of deionized water (1 mL), the lipid film was incubated for 1 h at 40 °C under gentle agitation. The obtained lipid dispersion was filtered four times through a syringe equipped with a 30-μm nylon net filter (Merck Millipore, Billerica, MA, USA). We prepared a lipid dispersion of 1-azidohexane (9 mM) and cholesterol (1 mM) by dissolving these lipids in chloroform using an amber glass vial. A film was formed by evaporation of the solvent. The residual chloroform was removed as described above. After addition of deionized water (1 mL), the lipid film was incubated and the lipid dispersion filtered as describe above. The lipid dispersions (300 μL each) were mixed for 10 h, which resulted in the formation of numerous GVs (>10 μm diameter). Stained GVs were prepared by mixing stock solutions of AH/Chol (9/1 mM) and Texas Red-DHPE (0.02 mol%) in an amber glass vial. Finally, the GVs were diluted with 600 μL of deionized water and filtered four times with a 30-μm nylon net filter.

### Transmission electron microscopy

One drop (5 µL) of a mixed lipid dispersion of LH (4.5 mM) and AH/Chol (4.5/0.5 mM) was placed on a Formvar film formed on a cupper mesh (grid size: 200 × 200 µm) after carbon deposition and plasma hydrophilization of the film (JEOL, Japan). After incubation under atmospheric pressure for 1 minute, the excess dispersion was drawn off with a filter paper. Then one drop (5 µL) of uranium acetate aqueous solution (1.5%) was placed on the mesh and incubated for 1 minute and the excess solution was drawn off. This staining process was repeated three times and finally the mesh was dried for 5 minutes under atmospheric pressure. The mesh was examined using an H-7500 transmission electron microscope (HITACHI, Japan).

### DNA encapsulation

Lipid dispersions of LH and AH/Chol were mixed for 24 h with a solution containing lambda phage DNA (7 nM), SYBR Green I (1/100), and Tris-EDTA Buffer (10×) at room temperature. After encapsulation, the GVs were diluted with 2400 μL of deionized water, filtered four times with a 30-μm nylon net filter, and collected for microscopic observation.

### RhodamineB-tagged dextran encapsulation

Lipid dispersions of LH and AH/Chol were mixed for 48 h with a solution containing RhodamineB-tagged dextran (0.05 mM) and deionized water at room temperature. After encapsulation, the GVs were diluted with 1200 μL of deionized water, filtered four times with a 30-μm nylon net filter, and collected for microscopic observation.

### Preparation of samples and microscopic observation

Phase contrast and fluorescence microscopy, as well as video imaging of growth and fission processes, were conducted using an IX71 inverted phase contrast microscope (Olympus, Tokyo, Japan) equipped with a 40× objective lens, fluorescence filters (λ_ex_; 460–495 nm, λ_em_; 510–550 nm (WIBA) for SYBR Green I, λ_ex_; 565–585 nm, λ_em_; 600–690 nm (FMCHE) for TexasRed, and λ_ex_; 565–585 nm, λ_em_; 600–690 nm (FMCHE) for RhodamineB) and a DP72 CCD camera (Olympus). When we observed GVs containing both SYBR Green I (staining DNA) and TexasRed-DHPE (staining vesicular membrane) simultaneously, we used an Opto-line double band excitation and emission filter (λ_ex_; 460–498 nm and 571–598 nm, λ_em_; 509–538 nm and 611–644 nm). A specimen for microscopic observation was prepared as follows. On a cover glass with an adhered spacer (15 × 15 mm, 65 μL - Frame Seal Chamber; Bio-Rad, Hercules, CA, USA) the dispersions of immature mother GVs (10 μL), LH (10 μL), AH/Chol (10 μL), and the catalytic solution (10 μL) were placed next to each other forming a square. A cover glass was placed on top of the spacer to mix and seal the specimen. All experiments were performed at 22–25 °C. Each movie was captured at a time interval of approximately 100 sec.

### Image analysis

Images of growing and budding mother GVs taken from movie data were analyzed by ImageJ software (NIH, Bethesda, MD, USA) and a custom-made software written in Python. First, in each image the contours of a budding mother GV were fitted as two superimposed circles using ImageJ. Next the radii and the position of centers of the two circles were recorded. This process was repeated five times for each image and the averaged values were used for the following calculations using the Python software: surface area, volume of the whole GV, angle between two tangents at the neck and distances between the centers of those two circles. The images of each movie data were selected every 5 or 10 frames (approximately 0.3 sec intervals). When the focal plane of the image was judged as inaccurate for visual fitting by the ImageJ software, we omitted the image. All of the calculated values were plotted with their average values to show the absence of multimodality in noise distribution in Figs 3, S9, S16, and S17. We did not examine the data obtained by the image analysis of the mother GVs showing rotation or complete division because the focal plane tracing such GVs was difficult to establish.

### ^1^H NMR analysis

GV dispersions were freeze-dried to remove water and remaining AH following the addition of deuterated dimethylsulfoxide. In a preliminary reference experiment, we confirmed the absence of AH peaks in a freeze-dried sample of AH-Chol. The hydrogen atoms of the methyl group of cholesterol, where the carbon atom is numbered 18, were assigned to a singlet peak at δ 0.64. There was no shift of this singlet peak in all the samples obtained. Moreover, no other compounds, including ascorbic acid derivatives produced by the addition of copper, had peaks in this range. Therefore, we used this singlet peak as the internal standard to measure LH and TPC. We obtained several peaks at δ 7.85–7.89 and assigned them to the hydrogen atoms of the free triazole ring and the copper-bounded triazole ring produced by the CuAAC reaction. The broad peak observed at δ 2.79–2.84 was assigned to the hydrogen atom of the ethynyl group of LH. Various peaks were found on the chemical shift scale in the chemical analysis of the freeze-dried catalytic solution (i.e. ascorbic acid and copper chloride). However, none of them appeared on the ranges mentioned.

## Electronic supplementary material

Movie S1

Movie S2

Movie S3

Movie S4

Supplementary Info
