# Supplementary material for: Budding and Division of Giant Vesicles Linked to Phospholipid Production
Source: Sci Rep. 2019 Jan 17;9:165. doi: 10.1038/s41598-018-36183-9 (PMC6336860; doi:10.1038/s41598-018-36183-9)
Supplement: Supplementary file 5 — Supplementary Info [file 41598_2018_36183_MOESM5_ESM.pdf]

Supplementary Information

Budding and Division of Giant Vesicles Linked to Phospholipid Production

Juan M. Castro[1], Hironori Sugiyama[1], and Taro Toyota[1, 2,] *

[1]Department of Basic Science, Graduate School of Arts and Sciences, The University of Tokyo, 3-8-1 Komaba, Meguro-ku, Tokyo 153-8902, Japan

[2]Universal Biology Institute, The University of Tokyo, 3-8-1 Komaba, Meguro-ku, Tokyo 153-8902, Japan

Supplementary Information Index

Figures … p.2
Table …p.19
Video clip descriptions …p.20

Figures

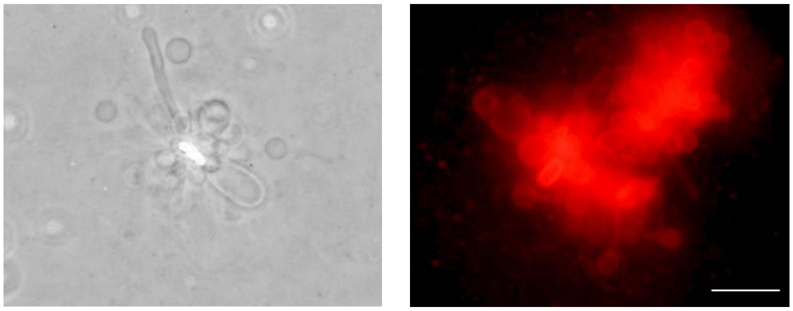

Figure S1. Formation of immature mother GVs by lipid dispersion mixture and *in situ* self-assembly. Left: Phase contrast micrograph. Right: Fluorescence micrograph. These images were taken 1 h after the mixing of LH (9 mM) and AH/Chol (AH/Chol, 9/1 mM) labeled with 0.02 mol% TexasRed-DHPE. Scale bar, 20 μm.

(A) **(B)**

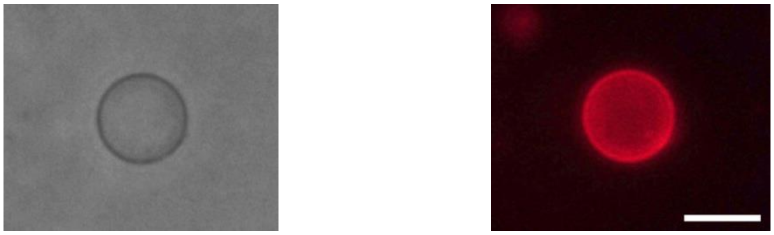

(C)

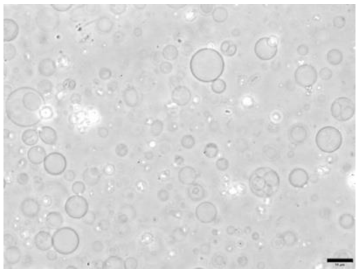

Figure S2. Micrographs of giant phospholipid vesicles (GVs) composed of LH, AH and cholesterol (4.5/4.5/0.5 mM) labeled with 0.02 mol% TexasRed-DHPE. (A) Phase contrast micrograph and corresponding fluorescence image (B) of a GV. (C) Phase contrast micrograph of GVs in a wide field of view. These images were taken 24 h after the mixing of LH (9 mM) and AH/Chol (AH/Chol, 9/1 mM) labeled with 0.02 mol% TexasRed-DHPE. Scale bars = 10 μm.

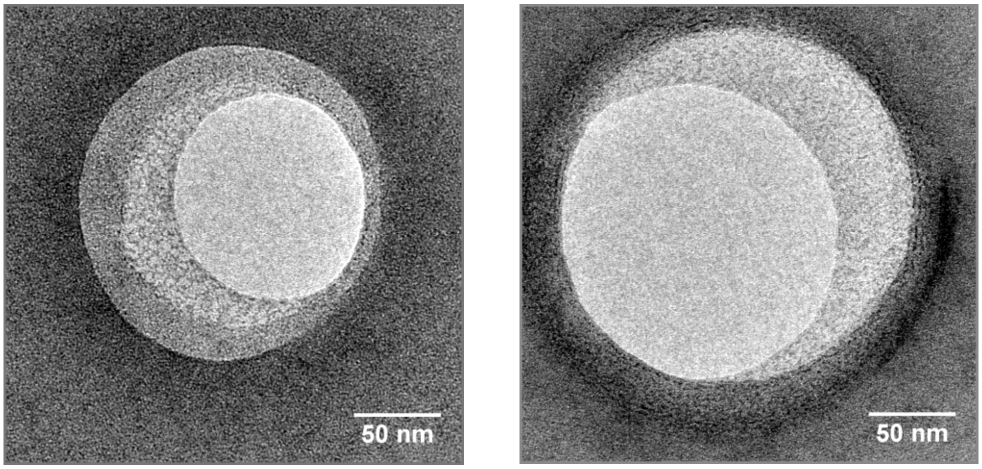

Figure S3. Transmission electron micrographs of vesicular membranes observed in an immature mothers' dispersion sample. Vesicles with a diameter larger than 1 μm were not observed in the sample, plausibly because these giant vesicles collapsed during the drying process.

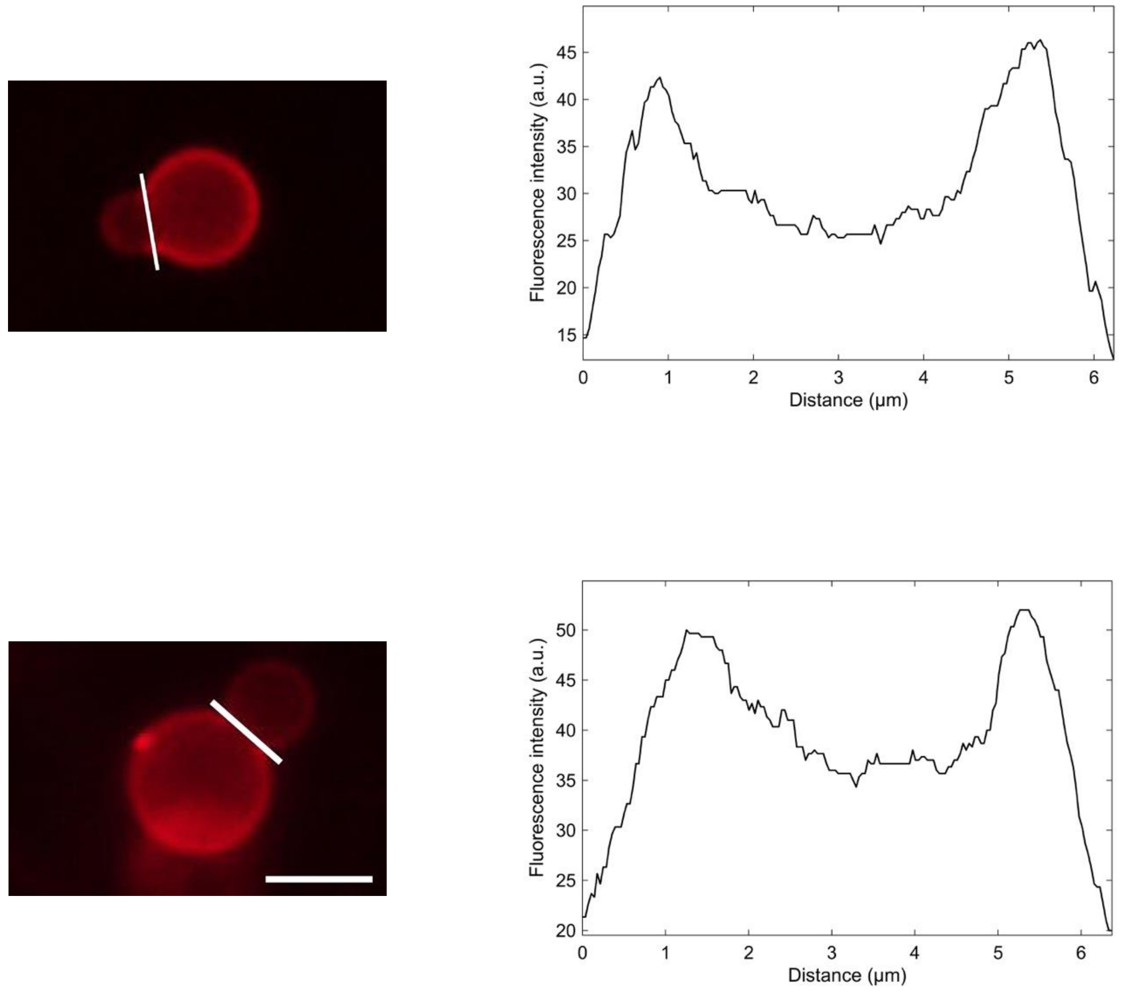

Figure S4. Fluorescence micrograph of giant vesicles during budding. The left panels display pear-like structures with a wide neck. The right panel display corresponding fluorescence intensity profiles along the lines indicated in the images. Scale bar, 10 μm.

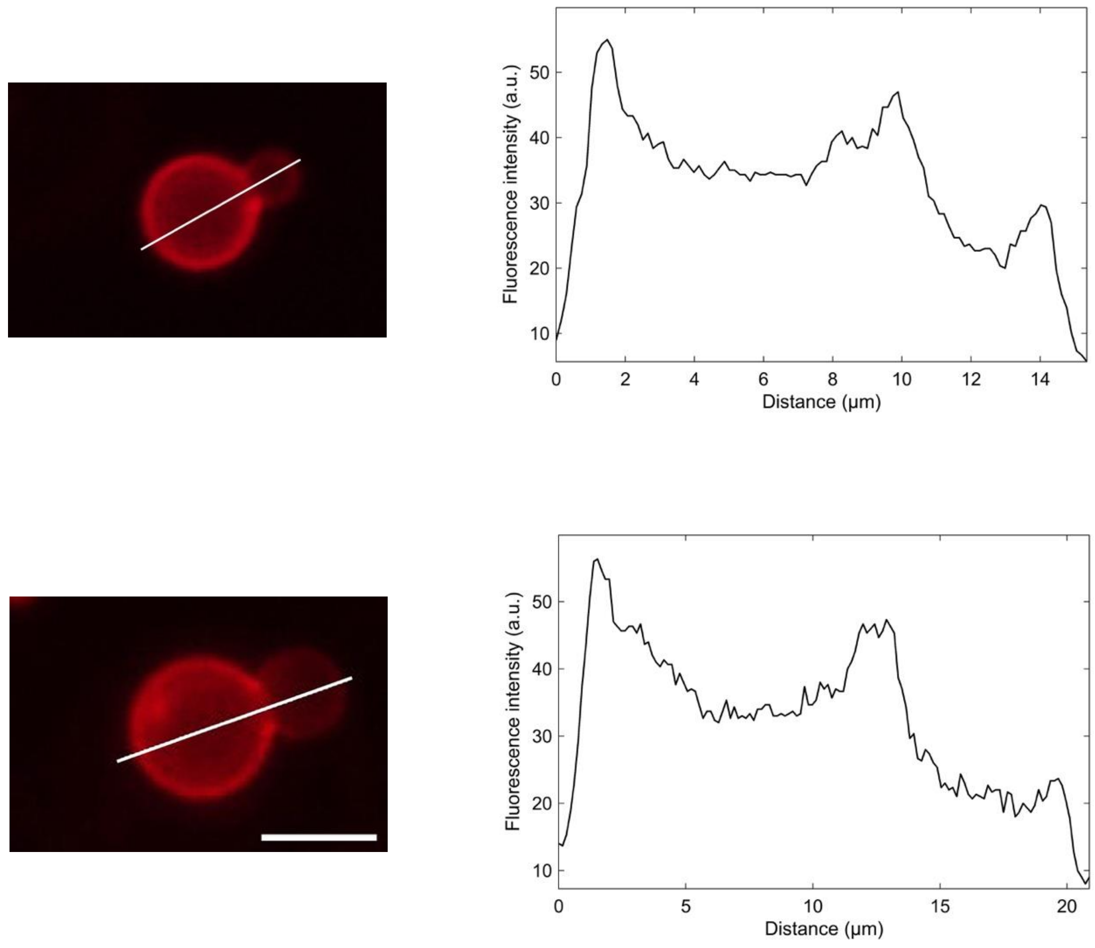

Figure S5. **Fluorescence micrographs showing the asymmetrical distribution of the fluorescent probe (Texas-Red DHPE) between mother and daughter vesicles.** The left panels display giant vesicles labeled with 0.02 mol% Red-DHPE. The right panels display the corresponding fluorescence intensity profiles along the lines indicated in the images. Scale bar, 10 μm.

(A) (B) (C) (D)

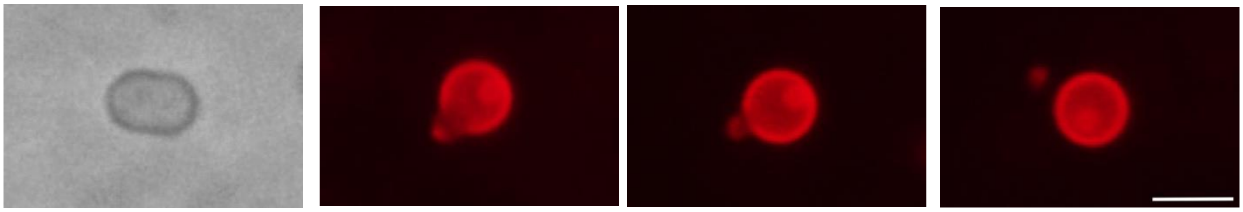

Figure S6. Budding and division of a non-spherical mature giant vesicle after the addition of a catalytic solution and reactive precursors. Selected frames from a phase contrast and fluorescence microscopy video (t = 0 sec). Images were acquired at: (A) 45:29 sec, (B) 47:36 sec, (C) 52:03 sec, and (D) 58:04 sec. Scale bar, 10 μm.

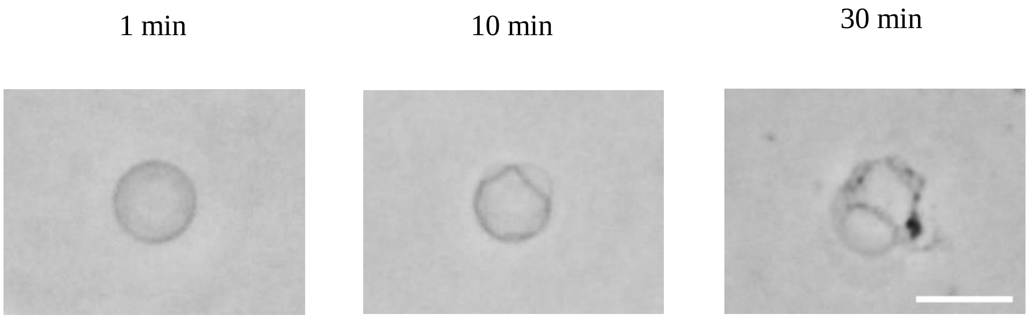

Figure S7. Phase contrast micrographs of an immature mother vesicle after the addition of sodium chloride (10 mM), ascorbic acid (20 mM), and deionized water (*t* = 0 min). Scale bar, 10 μm.

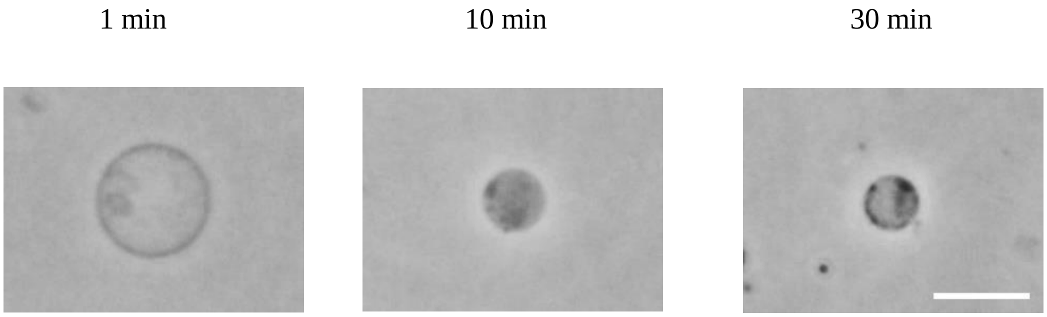

Figure S8. Phase contrast micrographs of a giant vesicle after the addition of lipid dispersions of LH (9 mM) and AH/Chol (AH, 9 mM; Chol, 1 mM) with sodium chloride (10 mM), ascorbic acid (20 mM), and deionized water at 0 min. Scale bar, 10 μm.

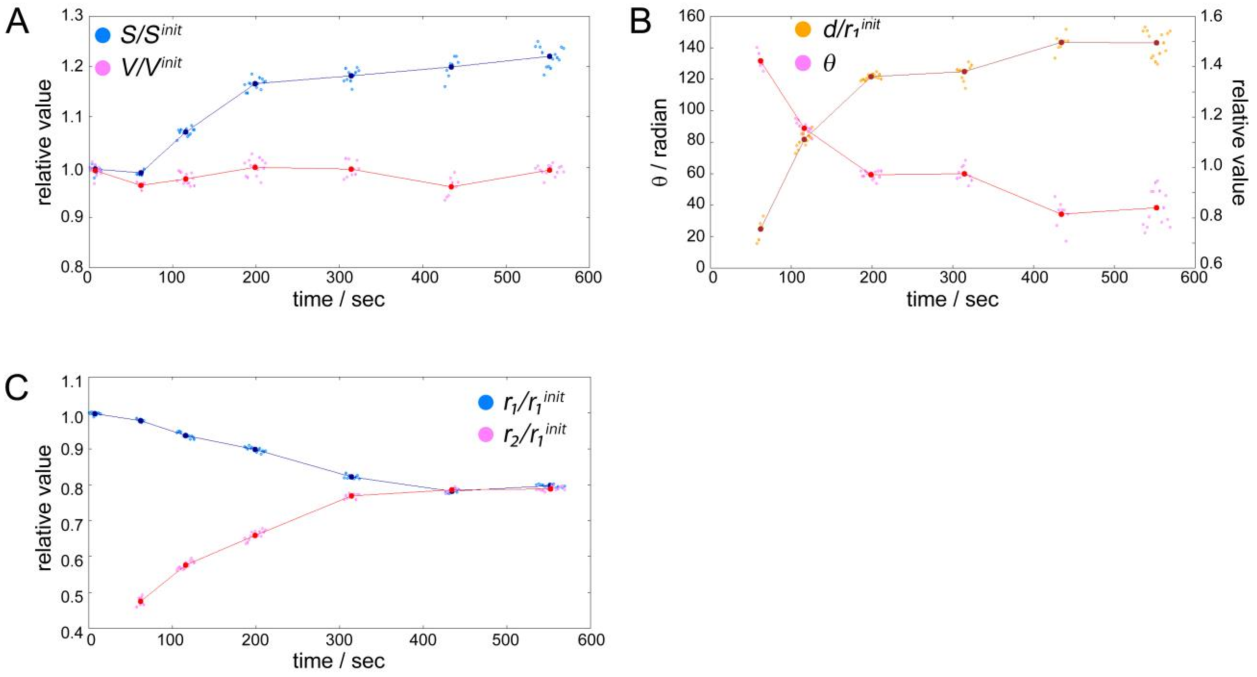

Figure S9. Time course of relative volume and surface area of a mature mother GVs during budding and its cross-section geometrical parameters (A) The average values of relative surface area (blue) and volume (magenta) of an individual mother GV during budding. The pale blue and magenta dots represent all the data obtained by the analysis of sequential snapshots. (B) The angle (red dots), the distance of centers of Sec. 1 and Sec. 2 (brown dots). (C) The relative radii of Sec. 1 (blue dots) and Sec. 2 (magenta dots). The initial radius of Sec 1 was assigned as 1 for these relative radii. The pale-colored dots represent all the data obtained by the analysis and the intensely colored dots represent the average of the data set form snapshots taken from one movie.

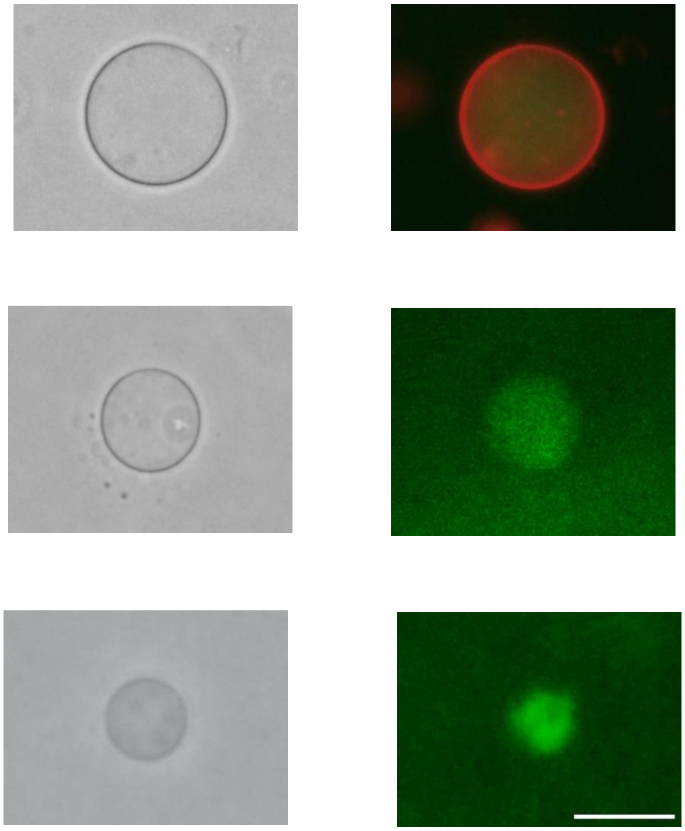

Figure S10. Lambda phage DNA-containing immature mother giant vesicles (GVs). LH/AH/Chol (4.5/4.5/0.5 mM) GVs encapsulating SYBR green I labeled-DNA. The left panels display phase contrast micrographs. The right panels display the corresponding fluorescence micrographs. The GV in the top-right panel was labeled with 0.02 mol% TexasRed-DHPE. Scale bar, 10 μm.

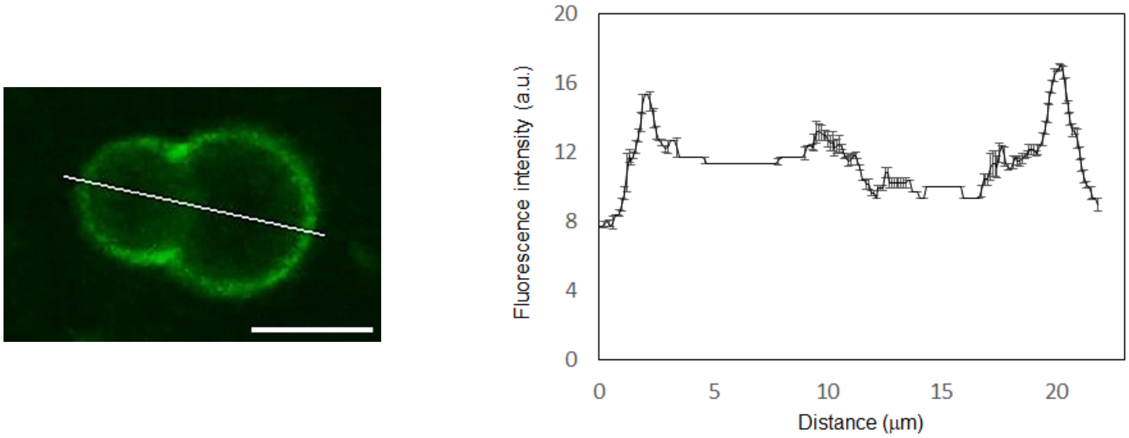

Figure S11. Fluorescence micrographs of a mother giant vesicle (GV) containing lambda phage DNA after the addition of the catalytic solution and membrane precursors. DNA was labeled with SYBR Green I. The left panel displays the relocation of nucleic acids at the vicinity of the inner surface of the GV. The right panel is the corresponding fluorescence intensity profile along the line indicated in the image. The standard deviation bars of the fluorescence intensities along the line were calculated from five sequential snapshots of the corresponding mother GV captured at time interval of 0.2 sec. Scale bar, 10 μm.

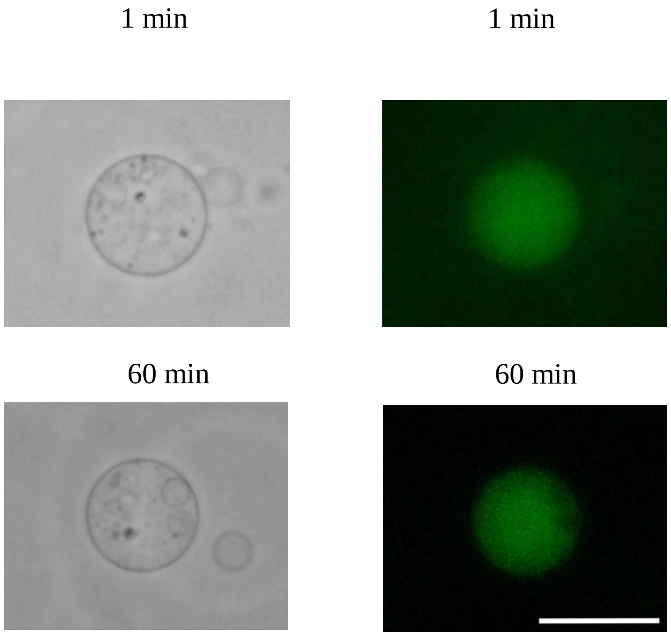

Figure S12. Lambda phage DNA-containing GVs exposed to membrane precursors without the catalytic solution for 1 h. The encapsulated DNA remained visible in the entire inner space of the vesicle without relocation. Scale bar, 10 μm.

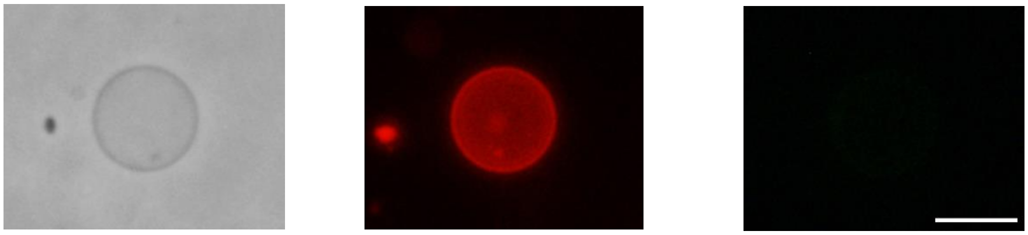

Figure S13. Immature mother vesicle containing SYBR Green I without lambda phage DNA. The left panel displays a phase contrast micrograph. The center panel displays the corresponding fluorescence image. The vesicle was labeled with 0.02 mol% TexasRed-DHPE. The right panel demonstrates that green fluorescence was not observed in the specimen. Scale bar, 10 μm.

(A) 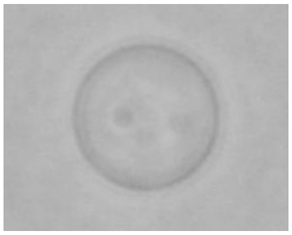

(B) 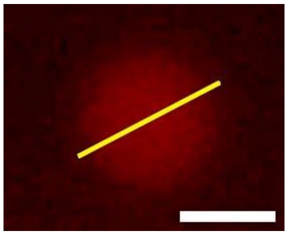

(C) 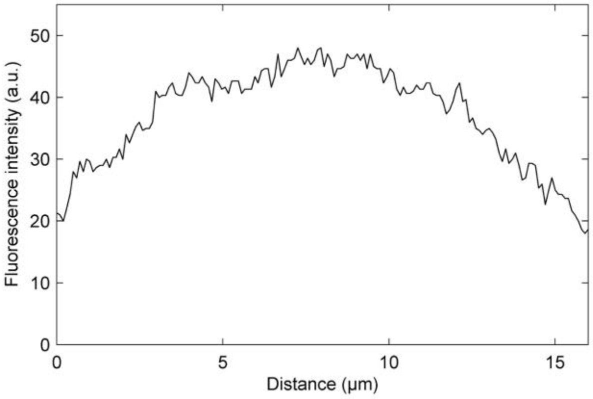

Figure S14. Rhodamine B-tagged dextran containing immature mother vesicle (GV). LH/AH/Chol (4.5/4.5/0.5 mM) GV encapsulating 0.05 mM Rhodamine B-tagged dextran (molecular weight = 10000). (A) Phase contrast micrograph. (B) Corresponding fluorescence micrograph. (C) Corresponding fluorescence intensity profile along the yellow line indicated in panel B. Scale bar, 10μm.

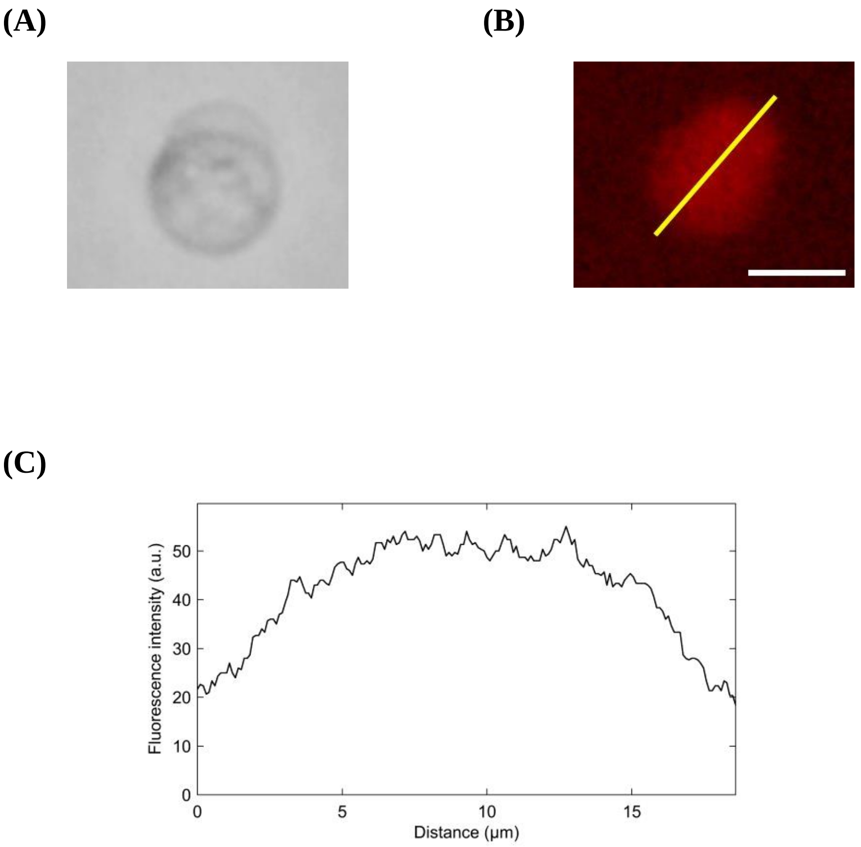

Figure S15. Mature mother vesicle and its bud encapsulating Rhodamine B-tagged dextran after the addition of the catalytic solution and membrane precursors. Selected frames from a phase contrast and fluorescence microscopy video (t = 0 sec). Images were acquired at: (A) 23:34 sec, and (B) 24:26 sec. The fluorescence micrograph shows the homogenous distribution of Rhodamine B-tagged dextran in the entire inner space of the mother vesicle and its bud after chemical induction. (C) Corresponding fluorescence intensity profile along the yellow line indicated in panel B. Scale bar, 10 μm.

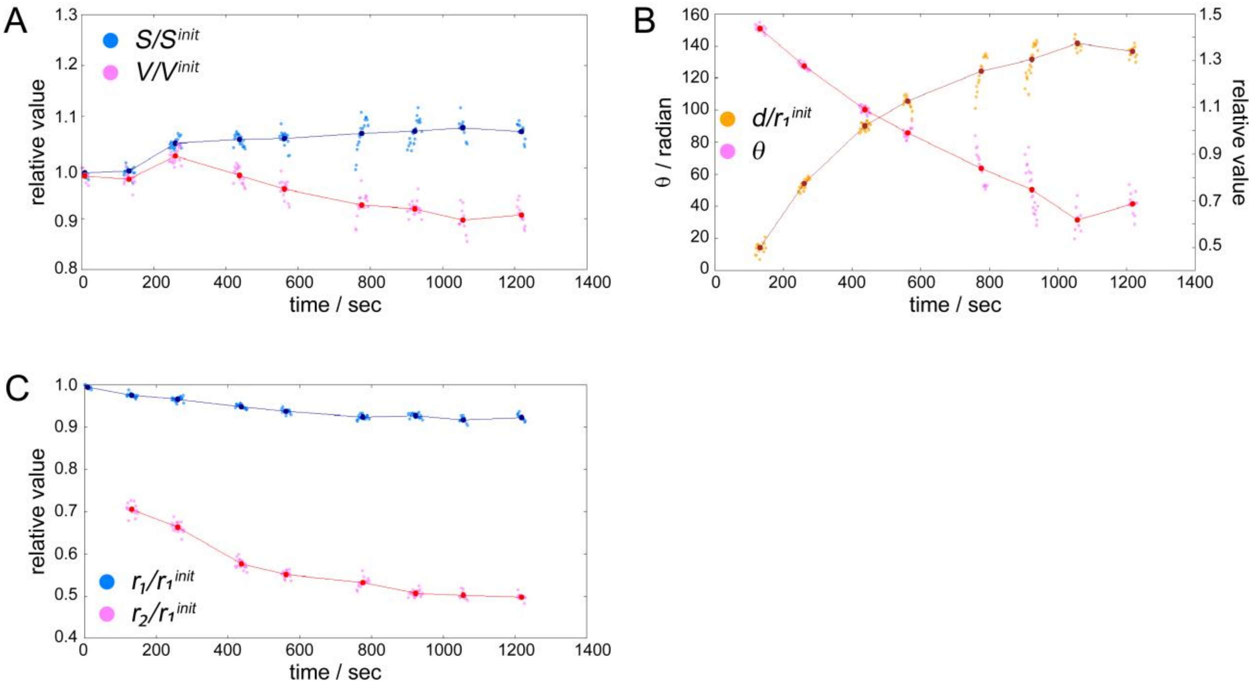

Figure S16. Time course of relative volume and surface area of a mature mother GV during budding and its cross-section geometrical parameters (A) The average values of relative surface area (blue) and volume (magenta) of an individual mother GV during budding. The pale blue and magenta dots represent all the data obtained by the analysis of sequential snapshots. (B) The angle (red dots), the distance of centers of Sec. 1 and Sec. 2 (brown dots). (C) The relative radii of Sec. 1 (blue dots) and Sec. 2 (magenta dots). The initial radius of Sec 1 was assigned as 1 for these relative radii. The pale-colored dots represent all the data obtained by the analysis and the intensely colored dots represent the average of the data set form snapshots taken from one movie.

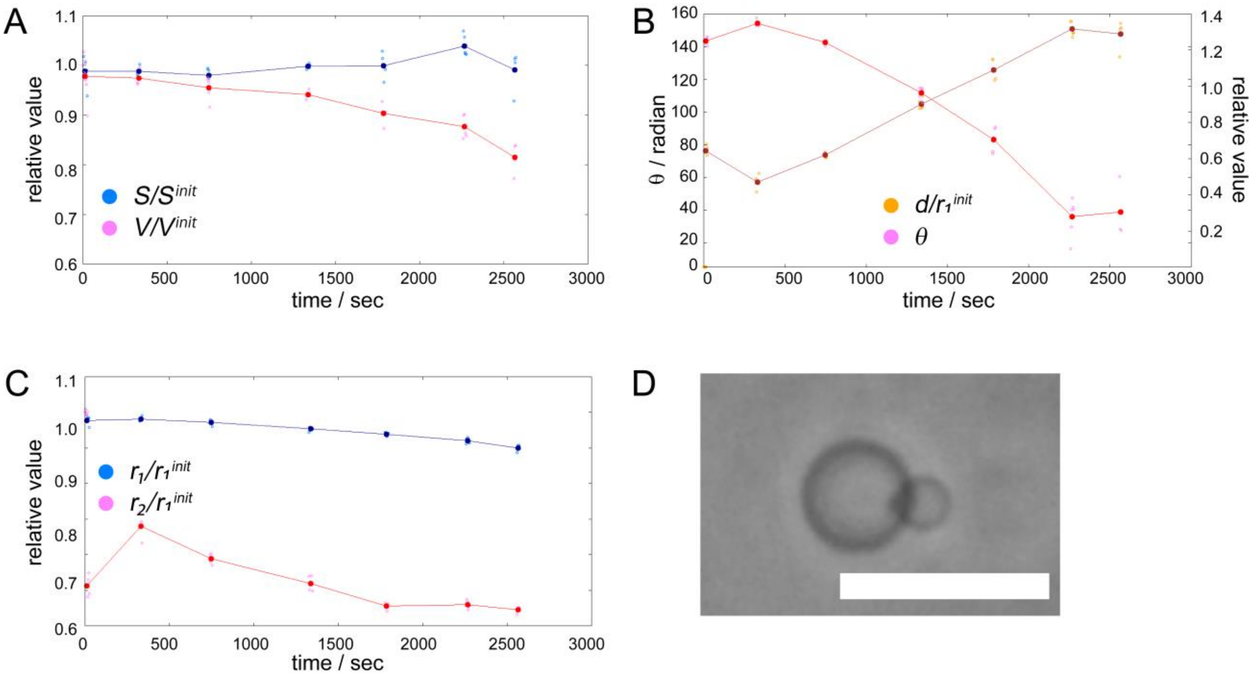

Figure S17. Time course of relative volume and surface area of a mature mother GV during budding following the adhesion of its daughter and its cross-section geometrical parameters (A) The average values of relative surface area (blue) and volume (magenta) of an individual mother GV during budding. The pale blue and magenta dots represent all the data obtained by the analysis of sequential snapshots. (B) The angle (red dots), the distance of centers of Sec. 1 and Sec. 2 (brown dots). (C) The relative radii of Sec. 1 (blue dots) and Sec. 2 (magenta dots). The initial radius of Sec 1 was assigned as 1 for these relative radii. The pale-colored dots represent all the data obtained by the analysis and the intensely colored dots represent the average of the data set form snapshots taken from one movie. The bud formed by the mother became a daughter vesicle and then the born daughter partly adhered to the mother (see (D), bar = 20 μm), resulting in the decrease of S/S^{int} from 1.04 to 1 at 2550 sec. Note that the data sets of the relative surface area of the GV at 2250 sec were statistically distinguishable from the first data sets, while the final data sets at 2550 sec were statistically indistinguishable.

Table S1. Integral value of peaks appearing in ^{1}H NMR charts of GV dispersions

Sample	Integral value at δ 7.85-7.89 (singlet; triazole ring)	Integral value at δ 2.79-2.84 (triplet; ethynyl group)
Immature mother GVs (20 min after preparation)	3.44	35.23
Mature mother GVs (1 min after mixing immature mother GVs, LH, AH/Chol, and catalytic solution)	5.84	33.32
Mature mother and daughter GVs (1 hr after mixing immature mother GVs, LH, AH/Chol, and catalytic solution)	11.01	103.13

A singlet peak at δ 0.64 was assigned as three as an internal standard because this singlet peak corresponds to the hydrogen atoms of the methyl group of cholesterol, which is chemically stable and non-volatile material upon freeze-drying in the examined GV dispersions.

Movie clip descriptions

Four movie clips are attached to show the shape transformations, division, and DNA transport of GVs in the presence of the catalytic solution and the membrane precursor.

Movie S1: Phase contrast microscopy video showing the budding and division of a mother GV driven by the addition of a catalytic solution and reactive precursors. Scale bar, 10 μm.

Movie S2: Phase contrast microscopy video showing the budding and division (without detachment) of a mother GV driven by the addition of a catalytic solution and reactive precursors. Scale bar, 10 μm.

Movie S3: Phase contrast and fluorescence microscopy video showing the localization and transport of lambda phage DNA molecules from a mature mother vesicle to its bud. Scale bar, 10 μm.

Movie S4: Phase contrast microscopy video showing the budding and division of another mother GV driven by the addition of a catalytic solution and reactive precursors. Scale bar, 10 μm.
